# *Lactobacillus gasseri* Suppresses the *Helicobacter pylori*-Induced Hummingbird Phenotype by Inhibiting CagA Phosphorylation and SHP-2 Interaction

**DOI:** 10.3390/ijms26146718

**Published:** 2025-07-13

**Authors:** Rajesh K. Gupta, Tanvi Somiah, Amelia C. Steinlein, Ann-Beth Jonsson

**Affiliations:** Department of Molecular Biosciences, The Wenner-Gren Institute, Stockholm University, SE-10691 Stockholm, Sweden; rajesh29177@gmail.com (R.K.G.); mtanvisomiah@gmail.com (T.S.); amelia.steinlein@gmail.com (A.C.S.)

**Keywords:** *Lactobacillus*, CagA, *Helicobacter pylori*, cell morphology

## Abstract

*Helicobacter pylori* infection is the strongest known risk factor for the development of gastric cancer. The bacterium leverages several unique virulence factors to its advantage in order to colonize the human host. Among these, T4SS-delivered cytotoxin-associated gene A (CagA) has the most well-established links to severe forms of disease. To explore the effect of lactobacilli in disrupting CagA functions within host cells, we expressed HA-tagged humanized *cagA* in the human gastric epithelial AGS cell line and studied both the phosphorylation levels of CagA and its downstream binding partners. We found that gastric-specific *Lactobacillus gasseri* Kx110 A1 suppressed the phosphorylation of CagA and inhibited phosphorylation-dependent downstream signaling, resulting in the suppression of CagA-induced cell elongation of AGS cells, commonly known as the hummingbird phenotype. Surprisingly, phosphorylation-independent signaling was unaffected by *L. gasseri*. Furthermore, our confocal microscopy analysis revealed that CagA was mislocalized to the cytoplasm, suggesting that *L. gasseri* interferes with its membrane localization and thereby hinders its phosphorylation. Live *L. gasseri* that had direct contact with host cells was found to be necessary to suppress the hummingbird phenotype. In summary, the data suggest that a *L. gasseri* strain can inhibit CagA phosphorylation and suppress cell elongation.

## 1. Introduction

*Helicobacter pylori* is a highly successful gastric pathogen that inhabits about half the population of the world [1]. The bacterium is the causative agent of gastric diseases, including chronic inflammation, ulcers, gastritis, and mucosa-associated lymphoid tissue (MALT) lymphoma and adenocarcinoma [2]. Cytotoxicity-associated gene A (CagA), produced by *H. pylori*, is one of more than 30 genes expressed from the 40 kb DNA segment known as the cag pathogenicity island (*cag*PAI), and the role of CagA in virulence has been well-documented [3,4]. Several studies have demonstrated a strong link between CagA and disease severity; hence, the ability of *H. pylori* to produce CagA is considered a direct measure of its virulence.

CagA is translocated by *H. pylori* into host cells by the bacterial Type-4-secretion system (T4SS) secretion apparatus. Once inside the cells, CagA localizes itself to the inner surface of the plasma membrane by interactions with phosphatidylserine [5], where it subsequently undergoes tyrosine phosphorylation by host tyrosine kinases specifically at the Glu-Pro-Ile-Tye-Ala (EPIYA) motifs at the C-terminal [6,7]. Phosphorylated CagA then goes on to interact with several proteins, one of them being SHP-2 (Src homology-2 protein tyrosine phosphatase), which is a critical step for signaling to the actin cytoskeleton [8]. Normally, SHP-2 plays an important role in cell growth and motility [9,10]. Deregulation causes abnormal morphological changes in cells, characterized by dramatic cell elongation, which is aptly termed the hummingbird phenotype due to retraction defects [11]. Both membrane tethering of CagA and activation of SHP-2 are necessary to trigger the hummingbird phenotype, as can be seen in studies involving gastric cells transfected with CagA and SHP-2 mutant constructs, as well as by the addition of SHP-2-specific phosphatase inhibitor calpeptin to cells, both of which show failure to result in the hummingbird phenotype [12,13].

In addition to its phosphorylation-dependent effects, CagA can also regulate host signaling independently of its phosphorylation status. CagA binds to tight junction (ZO-1, JAM-A) and adherens junction proteins (E-cadherin, β-catenin), and its interaction with the polarity-regulating kinase PAR1b/MARK is especially important, as it interferes with microtubule organization and cell polarity [14]. These phosphorylation-independent interactions are crucial for disrupting epithelial cell polarity and compromising barrier integrity, contributing to disease progression. Extensive studies on CagA have revealed links to the activation of Rho GTPases, host cell actin cytoskeleton reorganization, the activation of protooncogenes or changes in their expression, the recruitment of transcription factor NF-kb, and the cytokine storm in response to infection [15,16,17].

Furthermore, in light of the urgent need for novel therapeutic approaches to *H. pylori* in response to antibiotic resistance, there is no specific treatment to date that is directed toward CagA. The use of lactobacilli as novel therapeutic agents, either on their own or in conjunction with antibiotics, is gaining traction. These bacteria form part of the normal gut microflora and combat a broad range of pathogens through competition for space, the release of anti-bacterial molecules such as bacteriocins, organic acids, and hydrogen peroxide, as well as by downregulation of pathogenic virulence factors or modulation of the host immune system [18]. They can counteract *H. pylori* infections by downregulation of the adhesion protein SabA, which results in reduced colonization [19]. On the host side, they are also capable of modulating cytokine responses, such as TNF, IL-6, and IL-8 production [20,21]. In this study, we investigated the effect of lactobacilli on CagA functions and identified the ability of *L. gasseri* Kx110 A1 to suppress the CagA-phosphorylation-mediated hummingbird phenotype, thereby paving the way for the development of a novel therapeutic agent against *H. pylori* infection.

## 2. Results

### 2.1. Suppression of the H. pylori-Mediated Hummingbird Phenotype by L. gasseri

**Figure 1:**
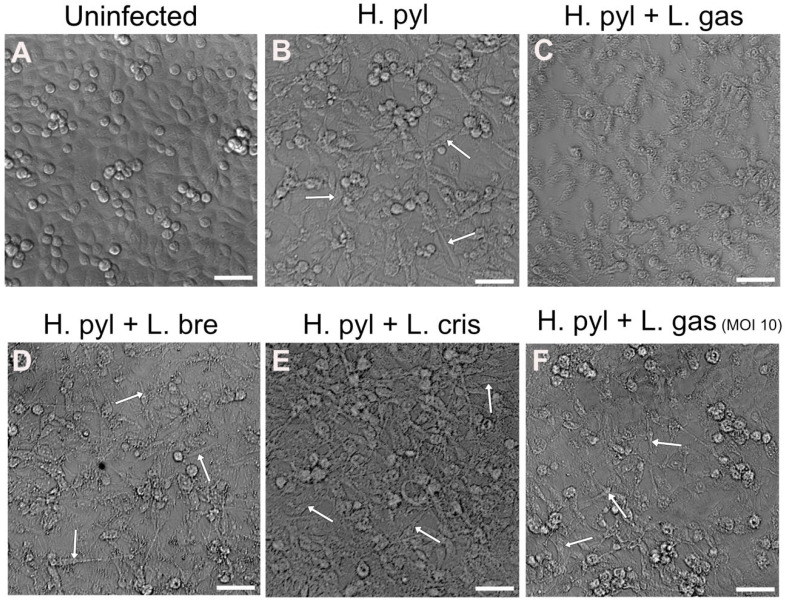
*L. gasseri* suppresses *H. pylori-* induced hummingbird phenotype in gastric epithelial AGS cells. Uninfected cells (**A**) and infected cells with *H. pylori* (**B**) at a MOI of 100 for 24 h, followed by 16 h treatment with *L. gasseri* (L. gas) (**C**), *L. brevis* (L. bre) (**D**), *L. crispatus* (L. crisp) (**E**) at a MOI of 50, and *L. gasseri* (L. gas) at a MOI of 10 (**F**). Differential interference contrast images of PFA-fixed AGS cells were captured with a Leica DMi8 microscope (Leica Microsystems, Wetzlar, Germany) using a 10× objective. White arrows mark the presence of elongated cells. Scale bar: 50 µm.

Changes in gastric cell morphology by *H. pylori* are well-studied and involve the CagA effector molecule, which is injected into host cells and confers dramatic cell elongation. To study whether lactobacilli can suppress the elongation of gastric cells into the so-called hummingbird phenotype, we infected gastric AGS cells with *H. pylori* for 24 h, followed by 16 h incubation with different *Lactobacillus* strains. We found that the strain *L. gasseri* Kx110 A1 (L. gas) was able to suppress cell elongation induced by *H. pylori* (Figure 1A–C). The ability to suppress the hummingbird phenotype was dependent on bacterial load, since *L. gasseri* at a lower MOI did not affect elongation (Figure 1F). Two other *Lactobacillus* strains, *L. brevis* (L. bre) and *L. crispatus* (L. cris), did not suppress cell elongation, demonstrating strain specificity (Figure 1D,E).

### 2.2. Lactobacillus Gasseri Suppress Elongation in CagA-Transfected Gastric Cells

**Figure 2:**
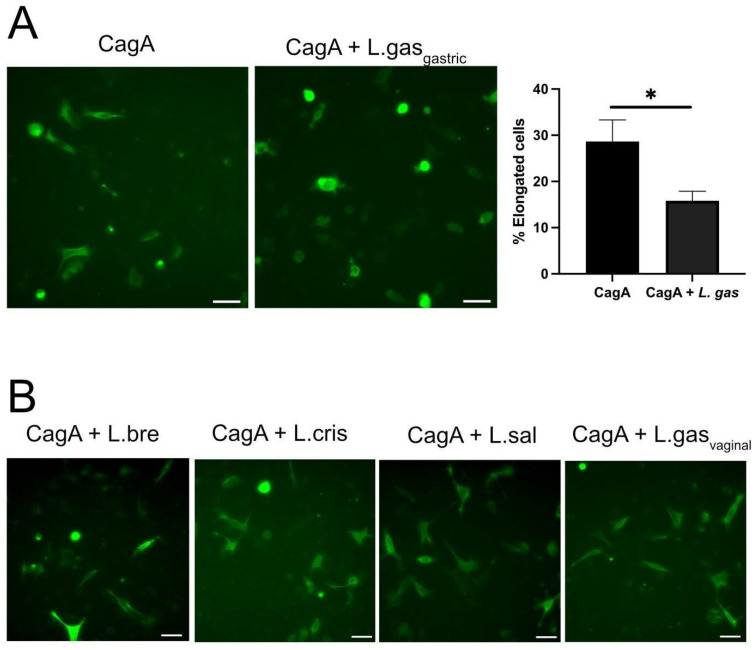
*L. gasseri* suppresses CagA-induced hummingbird phenotype in gastric epithelial AGS cells. Gastric AGS cells transfected for 48 h with a CagA construct (pcDNA3.1-CagA) were incubated with different *Lactobacillus* strains for 5 h. PFA-fixed cells were stained with rabbit-anti-HA antibodies to detect CagA, followed by anti-rabbit IgG Alexa Fluor 488, and imaged by a Leica DMi8 microscope using a 10× objective. (**A**) CagA-transfected cells were incubated with *L. gasseri* (CagA+L. gas) or with RPMI medium alone (CagA). The graph displays the percentage of elongated cells after counting at least one hundred cells, on three independent occasions. * *p* < 0.05, using a student *t*-test. (**B**) Treatment of CagA-transfected cells with *L. brevis* (L. bre), *L. crisptatus* (L. cris), *L salaviarus* (L. sal), or a vaginal *L. gasseri* strain (L. gas vaginal) for 5 h. Scale bars: 50 µm.

We have previously shown that some lactobacilli can inhibit the attachment of *H. pylori* to gastric cells by downregulating its virulence [19]. To rule out the possibility that the suppression of the hummingbird phenotype was a result of less CagA being translocated due to reduced *H. pylori* attachment, we used CagA-transfected cells instead of whole *H. pylori*. The incubation of CagA-transfected cells with *L. gasseri* efficiently suppressed the hummingbird phenotype, with a statistically confirmed reduced number of elongated cells (Figure 2A), which eliminated variation in bacterial adhesion as a possible mechanism. Incubation with *L. brevis*, *L. crispatus,* or *L. salivarius* (Figure 2B) did not suppress the elongation of the gastric cells. Further, a vaginally isolated *L. gasseri*-vag strain was incapable of bringing about an effect on altered cell shape (Figure 2B). These data demonstrate that the addition of a gastrically isolated *L. gasseri* strain to gastric cells with CagA-induced hummingbird phenotype significantly suppresses cell elongation.

### 2.3. Inhibition of Phosphorylation of CagA by L. gasseri Reduces CagA Interaction with the SHP-2 Phosphatase

**Figure 3:**
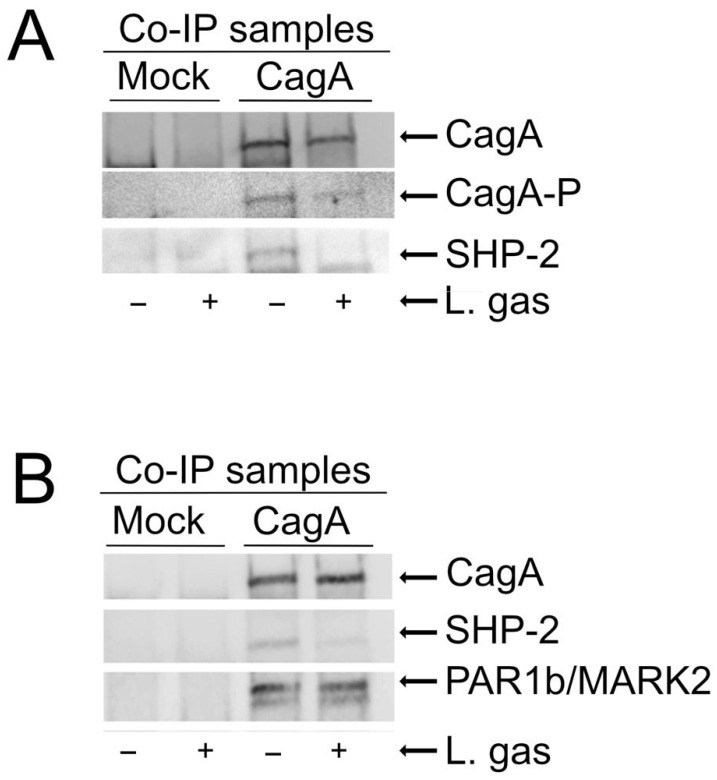
*L. gasseri* inhibits phosphorylation of CagA and reduces interaction with SHP2. AGS cells transfected with either the CagA construct or an empty vector (pcDNA3.1) were incubated for 5 h in RPMI medium, either with or without Lactobacillus gasseri (+). Cells were then lysed, and immunoprecipitation was performed using anti-HA antibodies to pull down CagA. Co-immunoprecipitated samples (Co-IP sample) were resolved on 4-20% precast polyacrylamide gels and transferred to nitrocellulose membranes. (**A**) CagA (anti-HA), CagA-P (anti-phospho-tyrosine), SHP-2 (anti-SHP2) (**B**) CagA, SHP-2, and Par1b/MARK2.

Elongation of cells requires phosphorylation of CagA [22]. To elucidate the mechanism behind the suppression of cell elongation by *L. gasseri*, CagA-transfected AGS cells were incubated with or without *L. gasseri* with empty vectors as a control. Phosphorylation of CagA in immunoprecipitated samples was then analyzed using anti-phosphotyrosine antibodies. We could observe suppression of phosphorylation of CagA in the *L. gasseri* samples compared to the untreated controls (Figure 3A). Phosphorylated CagA is known to interact with and activate the SHP2 phosphatase [11,12]. As expected, we found lower levels of SHP-2 in co-immunoprecipitated samples that were treated with *L. gasseri* compared to the untreated controls (Figure 3A). These data show that *L. gasseri* can suppress CagA-induced cell elongation by inhibiting the phosphorylation of CagA, leading to inhibition of CagA interaction with SHP-2 and blockage of SHP-2 activation, which is essential for cell elongation.

### 2.4. L. gasseri Does Not Interfere with Phosphorylation-Independent Functions of CagA

Since CagA is also known to mediate cytoskeletal changes independent of its phosphorylation status by interaction with PAR1/MARK kinases [14,23], we investigated whether *L. gasseri* could also affect this pathway. PAR1b/MARK2 expression in CagA-immunoprecipitated samples incubated with or without *L. gasseri* was analyzed, with an empty vector as control in both cases. Interestingly, we could not detect any differences in PAR1b/MARK2 levels in immunoprecipitated samples incubated with *L. gasseri*, as compared to those incubated with medium alone. Taken together, these results suggest that *L. gasseri* specifically suppresses the phosphorylation-dependent signaling of CagA (Figure 3B).

### 2.5. L. gasseri Disrupts the Membrane Localization of CagA

**Figure 4:**
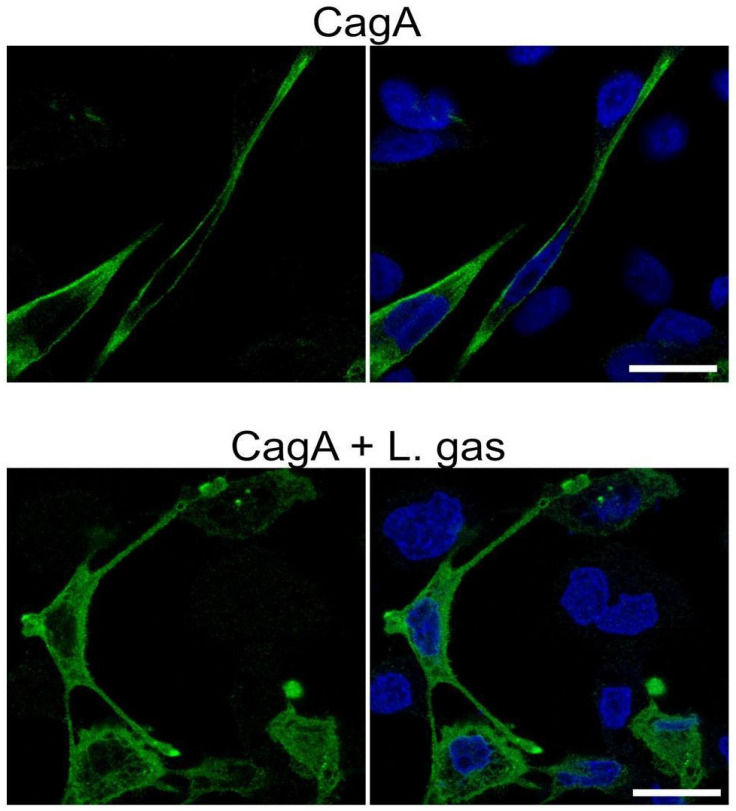
*L. gasseri* interferes with the membrane localization of CagA. Gastric AGS cells transfected with the CagA construct pcDNA3.1-CagA were incubated with *L. gasseri* (L. gas) at an MOI of 100 or RPMI medium alone for 5 h. Cells were then PFA-fixed, stained with anti-HA antibodies to detect CagA (green). DAPI (blue) was included in panels to the right to detect the nuclei. Stained cells were then analyzed by a Zeiss LSM 780 confocal microscope (Carl Zeiss AB, Oberkochen, Germany). Images represent a single z-section at the center of the cells. Scale bar: 20 µm.

Once translocated into host cells, CagA needs to be tethered to the inner plasma membrane in order to be phosphorylated by host kinases. Therefore, we carefully investigated whether *L. gasseri* disturbs the CagA membrane localization and hence hinders its ability to carry out its phosphorylation-dependent functions. CagA-expressing cells grown in chamber slides were analyzed by confocal microscopy after treatment with *L. gasseri*. In the control, i.e., untreated CagA-expressing elongated cells, CagA was strongly localized to the membranes. Interestingly, compared to untreated cells, CagA staining in *L. gasseri*-treated cells was diffused in the cytoplasm, suggesting that *L. gasseri* interfered with the localization of CagA to the membrane (Figure 4).

### 2.6. Live and Contact-Dependent Mechanism Underlies L. gasseri-Mediated Suppression of the Hummingbird Phenotype

**Figure 5:**
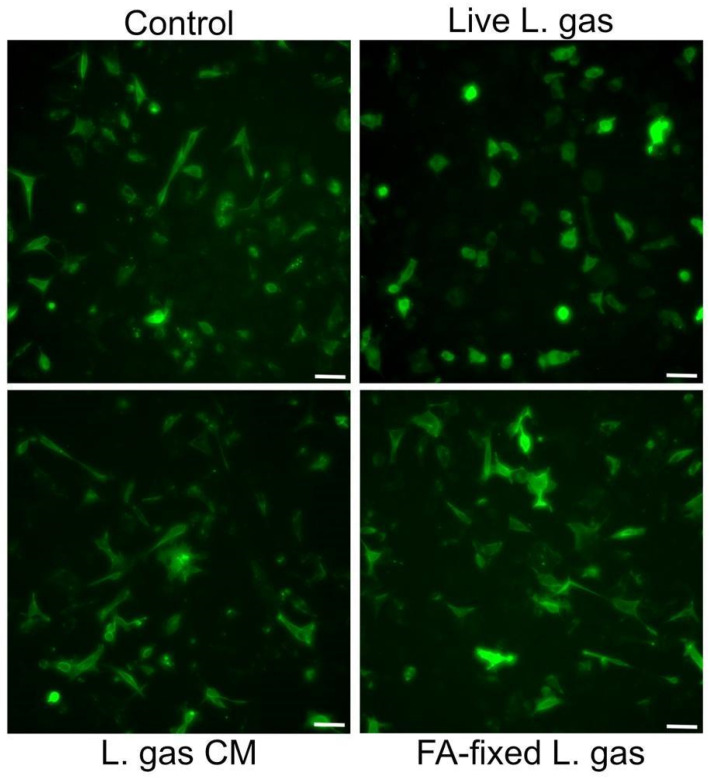
A live and contact-dependent mechanism underlies *L. gasseri*-mediated suppression of the hummingbird phenotype. Gastric AGS cells transfected with CagA construct pcDNA3.1-CagA were incubated with live *L. gasseri* (L. gas) at an MOI of 100, or with 4% PFA-fixed *L. gasseri* (FA-fixed L. gas), or with conditioned medium prepared from *L. gasseri* (L. gas CM) for 5 h. Images of PFA-fixed cells were then captured with a Leica DMi8 microscope (Leica Microsystems, Wetzlar, Germany) using a 20× objective. Scale bar: 50 µm.

In order to determine whether the active component(s) of lactobacilli were secreted/released into the supernatant or if live lactobacilli were needed for suppression of the hummingbird phenotype, we incubated CagA expressing cells with live or formaldehyde-fixed *L. gasseri,* or with *L. gasseri* conditioned medium, i.e., bacteria-free supernatants from *L. gasseri*. Interestingly, we observed that while cell elongation was reduced after incubation with live *L. gasseri*, neither formaldehyde-fixed *L. gasseri* nor the conditioned medium from the bacteria could mediate suppression of the hummingbird phenotype (Figure 5). These data suggest that the whole live *L. gasseri* triggers suppression of the hummingbird phenotype in gastric cells infected by *H. pylori*.

## 3. Discussion

The AGS cell line has been widely used as a model system to study CagA-induced effects, owing to its unique ability to undergo rapid and pronounced morphological changes upon ectopic expression of CagA. These morphological alterations, known as the “hummingbird” phenotype, are characterized by marked cell elongation and spindle-like protrusions. The distinct and easily observable nature of this phenotype makes AGS cells a valuable tool for monitoring CagA activity in both qualitative and quantitative assays. In addition, the hummingbird phenotype is a direct segue into oncogenic cell progression, since cells exhibiting this phenotype have increased cell motility and scattering, resembling epithelial–mesenchymal transition (EMT) [11].

Taking advantage of these characteristics, we established an assay using AGS cells to efficiently screen for *Lactobacillus* strains that can counteract CagA-induced morphological changes. Through a pilot-scale experiment, we identified the gastric-specific *Lactobacillus gasseri* Kx110 A1 strain as an effective suppressor of the CagA-induced hummingbird phenotype, establishing a foundation for large-scale screening of probiotics or potential therapeutic candidates. We assessed the ability of *Lactobacillus gasseri* to suppress the CagA-induced hummingbird phenotype in AGS cells by employing two complementary approaches: infection with CagA-positive *Helicobacter pylori* and direct ectopic expression of CagA via HA-tagged CagA plasmid transfection. The infection model mimics a more physiological context in which CagA is delivered to host cells by the *H. pylori* type IV secretion system at biologically relevant concentrations, while the transfection approach allows us to evaluate the direct effect of CagA in the absence of other bacterial components. In both approaches, *L. gasseri* significantly reduced the hummingbird phenotype, indicating it can interfere with CagA’s effects regardless of how it is introduced into the cells.

Since we observed a suppression of cell elongation by CagA in the presence of *L. gasseri*, we were interested in identifying the mechanism behind it. Unlike polarized epithelial cell lines such as MDCK, which require extensive remodeling of adherens junctions and cytoskeletal architecture, AGS cells lack well-defined cell polarity and tight junctions and do not express detectable levels of membrane-associated E-cadherin. These characteristics make AGS cells more permissive to rapid and pronounced morphological transformation into the highly elongated hummingbird phenotype upon CagA expression [24,25].

As a result, they are inherently more responsive to CagA-induced changes. However, the same properties reduce their reliance on CagA’s phosphorylation-independent mechanisms because AGS cells already exhibit a depolarized, junction-deficient phenotype. Consequently, the transformation in AGS cells is predominantly driven by phosphorylation-dependent pathways, particularly SHP2 activation and cytoskeletal reorganization. This implies that phenotypic screening assays using AGS cells are more likely to identify inhibitors that target CagA’s phosphorylation-dependent functions, while phosphorylation-independent activities may be underrepresented or overlooked. Consistent with this hypothesis, we found that the *L. gasseri* strain inhibited phosphorylation of CagA. The phosphorylated CagA-SHP-2 interaction could be considered the most crucial to fulfill *H. pylori*-induced changes in abnormal cell morphology and motility. SHP-2 consists of a pair of SH-2 domains at its N-terminal and a protein tyrosine phosphatase (PTP) at its C-terminal [26]. Phosphorylated CagA is capable of binding to the SH2 domain of SHP-2 and aberrantly activating it by maintaining it in a relaxed state; this exposes the PTPase domain, which dephosphorylates and inactivates key regulators of focal adhesion formation, affecting the control of normal cell morphology [13,27,28,29].

By co-immunoprecipitation experiments, we were able to observe that *L. gasseri* reduced the amount of phosphorylated CagA in cells, and consequently, the CagA interaction with SHP-2 was also lowered in cells treated with *L. gasseri* (Figure 3). SHP-2 is an oncoprotein owing to its strong ties with CagA-based carcinogenetic activity [30], and lowering its activity through *L. gasseri* could be a key point in preventing the conversion of a normal cell to an ultimately cancerous phenotype. The reduced level of phosphorylated CagA limits its interaction with the SHP-2 phosphatase, which is oncogenic in nature and promotes tumor growth.

CagA can also interact with PAR1b/MARK2 kinases independent of its phosphorylation status. Normally, PAR1b is involved in the maintenance and establishment of epithelial cell polarity by phosphorylating MAPs and destabilizing microtubules [31,32]. CagA is capable of directly binding and inhibiting PAR1b, thereby disrupting tight junctions and causing loss of apico-basal cell polarity. Notably, PAR1b also promotes CagA multimerization, which leads to stronger and more stable interactions with SHP2 [33]. Thus, the CagA-PAR1b interaction, although phosphorylation-independent, could also indirectly promote the hummingbird phenotype in infected cells [14,34]. To test whether *L. gasseri* was able to influence CagA activity independent of its phosphorylation, we looked at Par1b/MARK2 levels in CagA-transfected cells treated with *L. gasseri*, in comparison to cells treated with RPMI media as controls. SHP-2 expression was also checked in these experiments. Interestingly, we found that *L. gasseri* did not affect the interaction between CagA and PAR1b/MARK2, while SHP-2 showed lower levels of interaction in the *L. gasseri*-treated samples (Figure 4).

Since we saw that *L. gasseri* exclusively influenced CagA, we hypothesized that lactobacilli could perhaps interfere with the localization of CagA to the cell membrane. In order to interact with its downstream binding partner SHP-2, it is necessary for CagA to be first tethered to the membrane, where it undergoes phosphorylation by host kinases; only then can the phosphorylated CagA-SHP-2 complex form [35]. On the other hand, cytosolic, unphosphorylated CagA is sufficient to interact with PAR1b [36]. We used confocal microscopy to analyze the localization of HA-tagged CagA within the cells and observed that the staining was dispersed in the cytoplasm, in contrast to the membrane-associated localization of CagA seen in RPMI-treated controls (Figure 4), showing that *L. gasseri* mislocalized CagA within the cells and prevented it from anchoring to the cell membrane. Further, the incubation of CagA-expressing gastric cells with the *L. gasseri* strain interfered with CagA tethering to the membrane, as evidenced by its increased cytoplasmic staining and less membrane staining. By using CagA-transfected cells, our experimental model did not involve the *H. pylori* T4SS and its potential co-factors. These findings provide further support for studying the effect of *Lactobacillus* strains in the prevention and suppression of *H. pylori*-induced cell damage and highlight the importance of identifying effective *Lactobacillus* strains that might be used as treatments or preventive agents in the future.

In this study, we found that live lactobacilli in direct contact with the host cells were necessary to suppress CagA-induced cell elongation (Figure 5). These findings suggest that lactobacilli may bind to the host cell surface and activate a signaling pathway that disrupts the localization of CagA beneath the inner plasma membrane, possibly by blocking its interaction with phosphatidylserine or other membrane components. In the future, it would be interesting to identify and characterize the active component of the lactobacilli that confer this protective effect on the host. It would be motivating to validate these findings using animal or organoid models that more closely mimic physiological conditions, including the presence of mucus, microflora, and an acidic environment. It would also be valuable to investigate non-specific stress and immune responses of host cells, as well as to further characterize the *L. gassseri* Kx110 strain and compare it to other *Lactobacillus* species. At this stage, we do not know whether other *L. gasseri* isolates from the stomach may have similar properties. Future studies should focus on a more detailed characterization and whole-genome sequencing of the *L. gasseri* Kx110 A1 strain to identify potential genetic determinants responsible for its protective effects.

## 4. Materials and Methods

### 4.1. Bacterial Strains

The *Helicobacter pylori* strain 67:21 [1] was grown on Columbia blood agar plates (Thermo Fisher, Scientific, Waltham, MA, USA) supplemented with 8% defibrinated horse blood and 8% inactivated horse serum (Håtunalab, Uppsala, Sweden) for 3 days at 37 °C under microaerophilic conditions. The *Lactobacillus* strains used are described and listed in Table 1. Lactobacilli were grown on Rogosa agar plates and then cultured overnight in MRS broth (Oxoid Inc., Hampshire, UK) at 37 °C and 5% CO_2_ in a humidified environment. Prior to each experiment, overnight cultures of lactobacilli were washed and resuspended in RPMI 1640 (Thermo Fisher Scientific, Waltham, MA, USA), supplemented with 10% heat-inactivated fetal bovine serum (FBS, Sigma-Aldrich, Burlington, MA, USA).

### 4.2. Cell Culture

The gastric epithelial cell line AGS (ATCC, CRL-1739) was cultured in RPMI 1640 (Thermo Fisher, Scientific, Waltham, MA, USA) supplemented with 10% heat-inactivated fetal bovine serum (FBS, Sigma Aldrich, Burlington, MA, USA) and maintained at 37 °C and 5% CO_2_ in a humidified environment.

### 4.3. Plasmids and Transfection

For the transfection of AGS cells, the humanized *cagA* gene, along with a hemagglutinin (HA) tag, as previously described in Ohnishi et al., 2008 [2], was modified by the addition of HindIII and EcoRV restriction sites at the 5′ and 3′ ends, respectively. The customized constructs, cloned into pcDNA3.1(+) plasmid vectors (pcDNA3.1-CagA), were purchased from GenScript (Piscataway, NJ, USA). Empty pcDNA3.1(+) plasmid vectors (Genscript) were used as a mock transfection control. All constructs were transfected into AGS cells using Opti-mem (Thermo Fisher) and Lipofectamine 2000 (Thermo Fisher), as per the manufacturer’s instructions.

### 4.4. Infection of Gastric Cells with Whole H. pylori and Lactobacilli

Gastric AGS cells grown in 12-well plates were infected with *H. pylori* at an MOI of 100 for 24 h, followed by an overnight infection with lactobacilli at an MOI of 50 or 10, at 37 °C and 5% CO_2_ in a humidified environment. After overnight incubation, the cells were washed with PBS and fixed with 4% paraformaldehyde for 10 min. Differential interference contrast images of the fixed cells were collected using a Leica DMi8 microscope (Leica Microsystems, Wetzlar, Germany) with a 10× objective.

### 4.5. Infection of CagA-Expressing Gastric Cells with Lactobacilli

Gastric cells grown in 12-well plates were transfected with 1 µg of pcDNA3.1-CagA or empty pcDNA3.1 vectors and left for a further 48 h prior to infection with either lactobacilli at an MOI of 100 or RPMI media as a control, at 37 °C and 5% CO_2_ in a humidified environment. After 5 h of incubation, the cells were washed with PBS and fixed with 4% paraformaldehyde for 10 min and then analyzed by immunohistochemistry, as described below.

### 4.6. Immunocytochemistry and Confocal Microscopy

CagA-expressing AGS cells, treated with either lactobacilli or RPMI medium as a control, were paraformaldehyde-fixed, blocked with 5% BSA for 1 h at room temperature, and then treated with rabbit anti-HA (#3724, Cell Signaling Technology #3724, 1:1000) for 1 h at RT to detect CagA-HA. Cells were washed three times with PBST (PBS + 0.02% Tween20) containing 0.5% BSA and incubated with anti-rabbit IgG Alexa Flour 488 (A21206; Thermo Fisher) for 1 h at RT, washed three times with PBST again, and then finally incubated with DAPI solution (1 mM, Thermo Fisher) for 30 min at RT. Following the final three washes, 400 µL PBS was added to each well, and the cells were analyzed using a Leica DMi8 microscope for images. For confocal analysis, AGS cells were cultured on chamber slides, mounted using ProLong Gold Antifade Mountant with DAPI (Thermo Fisher), and imaged using a Zeiss LSM 780 confocal microscope (Carl Zeiss AB, Oberkochen, Germany).

### 4.7. Co-Immunoprecipitation

AGS cells in 6 cm dishes were transfected with 8 µg of pcDNA3.1-CagA or empty pcDNA3.1 vectors and left for a further 48 h prior to infection with either lactobacilli at an MOI of 100 or RPMI media as a control, at 37 °C and 5% CO_2_ in a humidified environment. After 5 h of incubation, cells were washed once with PBS, and the cell lysates of each sample were collected in 500 µL of IP lysis buffer (Thermo Fisher), supplemented with a 1× EDTA-free protease inhibitor cocktail (Roche, Basel, Switzerland), 2 mM Na_3_VO_4_, and 2 mM NaF (Sigma-Aldrich). Rabbit anti-HA (#3724; Cell Signaling Technology, 5 µL, 1:1000) was added to each sample and incubated overnight at 4 °C with gentle shaking. The next day, 25 µL of Protein A/G Magnetic Beads (Thermo Fisher) was added to each sample and incubated at 4 °C for 1 h, with gentle shaking. Sample-bound magnetic beads were then washed with IP lysis buffer three times and finally resuspended in 25 µL of 2× sample buffer (Bio-Rad) containing 5% B-mercaptoethanol (Sigma-Aldrich). The samples were stored at −20 °C until Western Blotting.

### 4.8. Western Blotting

Thawed samples were heated to 95 °C for 10 min, centrifuged for 1 min at 10,000× *g*, and then loaded onto 4–20% precast polyacrylamide gels (Bio-Rad). After separation, the proteins were transferred to a nitrocellulose membrane (Bio-Rad) using a semi-dry transfer system (Bio-Rad, Hercules, CA, USA). The blots were incubated for 1 h at room temperature in blocking buffer (Intercept Blocking Buffer, LI-COR, or 5% BSA), followed by overnight incubation at 4 °C with primary antibodies: HA-CagA (#3724T; Cell Signaling Technology, Danvers, MA, USA), anti-phosphotyrosine (ab10321; Abcam, Cambridge, UK), SHP-2 (sc-7384; Santa Cruz Biotechnology, Santa Cruz, CA, USA), and PAR1b/MARK2 (PA5-84966; Thermo Fisher). After three washes, the blots were incubated with secondary antibodies and detected using either the LI-COR Odyssey system or chemiluminescence (Bio-Rad, #1705062), depending on the type of secondary antibody used.

### 4.9. Fixation and Preparation of Conditioned Medium from L. gasseri

*L. gasseri* grown on Rogosa plates was inoculated in MRS broth and grown overnight at 37 °C. The following day, cultures were centrifuged at 4500× *g* for 10 min, resuspended in fresh MRS broth, and allowed to grow further for 2 h at 37 °C. The cultures were then re-centrifuged, and the resulting bacterial pellets were resuspended in RPMI 1640 to an optical density (OD) of 1.0 (5 × 10^7^ cfu/mL). For the preparation of fixed lactobacilli, the bacteria were spun down, and the pellets were treated with 4% formaldehyde for 10 min. They were washed three times with PBS and then resuspended in RPMI medium to a concentration of 5 × 10^7^ bacteria/mL. For the preparation of conditioned medium, the bacteria were further incubated in RPMI for 2 h and then filter-sterilized to collect the conditioned medium.

## Figures and Tables

**Table 1:** *Lactobacillus* strains used in this study.

Strain	Characteristics	Abbreviation	Source
*L. gasseri* Kx110 A1	Human gastric biopsy	L. gas	[19]
*L. gasseri* MV1	Human vagina	L. gas vaginal	[19]
*L. crispatus* MV24-1a	Human vagina	L. cris	[19]
*L. salivarius* LMG 9477	Human saliva	L. sal	[19]
*L. brevis* ATCC 14869	Human faeces	L. bre	[19]

## Data Availability

The datasets used and/or analyzed during the current study are available from the corresponding author upon reasonable request.
